# Nigerian Female with Skin Lesions in the Leg and Face: Herpetic Sycosis Folliculitis

**DOI:** 10.1155/2016/7948497

**Published:** 2016-11-27

**Authors:** Dominique Dilorenzo, Naganna Channaveeraiah, Patricia Gilford, Bruce Deschere

**Affiliations:** Orange Park Medical Center Family Medicine GME, 2021 Professional Center Drive, Suite 100, Orange Park, FL 32073, USA

## Abstract

Nongenital HSV 1 presents outside the mucus membrane. Our patient had unusual presentation that caused diagnostic dilemma. 30-year-old native Nigerian female coming with fiancée to the United States presented to our service one day after arrival through ER with a lesion on her right ankle. She was diagnosed with cellulitis, started on antibiotics, and admitted to hospital. She had fever of 39.1°C. Head and neck exam showed multiple sized lesions over tongue and palate and inner aspect of lower lip. Abdomen and genital exam was normal. Skin exam showed lesions over the face and lesions over the lateral aspect of the right leg. There was ulcerated lesion over the right lateral malleolus with surrounding erythema and edema. Her tests showed elevated ESR of 98; HIV test was negative; CT scan of the ankle showed no abscess or osteomyelitis. TB quantiferon was indeterminate; AFB stain and culture were negative; HSV IgM was elevated at 1 : 16; RPR was negative; ANA was negative; malaria screen was negative, and blood cultures were negative for bacteria, fungus, and virus. Debrided wound had no growth of bacteria or fungus or virus. This case illustrates the unusual presentation of the HSV1 outside the mucus membrane and how it can be confused with other conditions that required extensive tests. Therapeutic trail with antiviral medications resolved lesions over the leg and face.

## 1. Presenting History

A 30-year-old female native of Sokoto, Western Nigeria, coming to the United States with fiancée for the first time presented to us one day after arrival at the hospital emergency room with a painful ulcerated lesion on her right ankle [1–7].

## 2. History Review

According to the patient, she noticed this lesion over the right ankle 10 days ago; she visited her pharmacist and there was a questionable history of trauma. The pharmacist in Nigeria performed incision and drainage of the pus and removal of unhealthy tissue at the lesion and started her on Trypsin, Amoxicillin/Clavulanate, and unknown pain medication. The pain from right ankle persisted and she went to community hospital and was treated with wound cleaning and other antibiotics. Patient then noticed bullous lesions over face and enlarging mouth ulceration.

## 3. Review of Systems

The patient was positive for fever, mouth ulcers, and stress and negative for weakness in any limbs, cough, eye pain, blurry vision, and history of keloid.

## 4. Past Medical History

The past medical history was significant for sickle cell trait.

## 5. Family History

The family history was noncontributory.

## 6. Personal History

Patient lives with her fiancé and has never been pregnant. She is a nonsmoker and denies alcohol or illicit substance use. Vaccinations status is not clear. Patient had no known history of BCG vaccination.

## 7. Physical Examination

**Figure 1:**
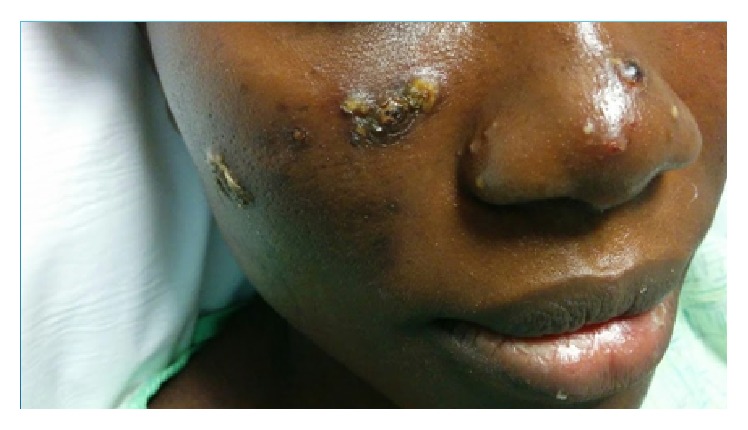


**Figure 2:**
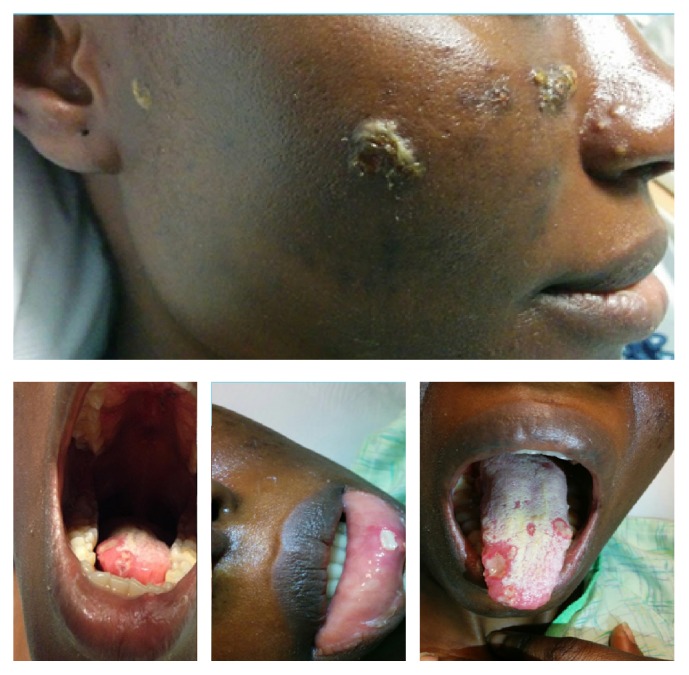


**Figure 3:**
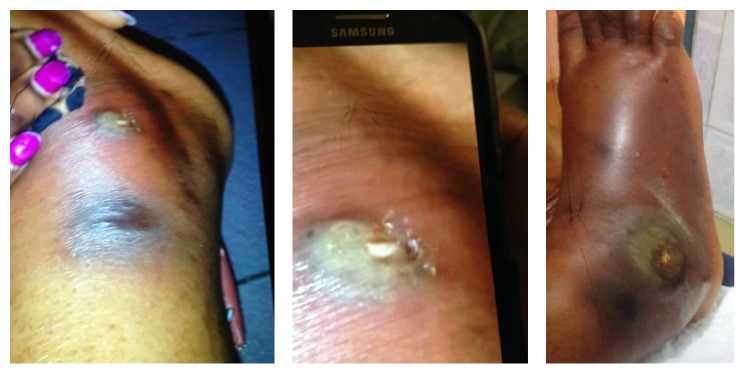


She is an African female. She was pleasant and in no distress. She had fever of 39.1°C, regular pulse of 73, BP of 121/74, and saturating 100% on room air. HEENT showed dry oral mucosa, multiple sized ulcerated lesions over tongue and palate and inner aspect of lower lip (Figure 2); no lymphadenopathy was found in neck, and the eye exam was normal. Lung and cardiovascular examinations were normal. Abdomen and genital exam was normal. Neuroexam was normal. Skin exam on the face showed multiple pustules, some with crusts and ulceration on elevated base measuring from 2 mm to 10 mm (Figure 1). One pustule measured 6 mm over the lateral aspect of the right leg. Ulcerated lesion was found over the right lateral malleolus measuring 3 cm × 4 cm with surrounding erythema and edema (Figure 3), and pulses were intact.

## 8. Investigations

CBC and BMP were within normal limits; ESR was elevated at 98 (normal rage 0–29 in females). HIV test was negative. X-ray of the ankle showed no bony abnormalities but a large soft tissue defect overlying the lateral malleolus with soft tissue swelling extending extensively along the dorsum of the foot. CT scan of the lower extremity and ankle showed no abscess or osteomyelitis. TB quantiferon was read as indeterminate high. Debrided tissue from the wound was tested with AFB stain. The acid-fast Bacilli (AFB) test was negative on the stain and culture. PCR was negative for* M. Ulcerans*. HSV IgM was elevated at 1 : 16 (normal; nonreactive); RPR was negative; ANA was negative; malaria screen was negative; and blood cultures were negative for bacteria, fungus, and virus.

## 9. Initial Admission Diagnosis and Hospital Course

The initial admission diagnosis of the patient was cellulitis. Infectious disease consultant was called in to help in the management as the history was not clear on the day of presentation to the hospital.

She was started on broad spectrum antibiotics in the ER (Vancomycin and Zosyn). She had the surgical debridement of the wound in the leg. After surgery, topical enzymatic debridement was done with Santyl to her foot wound and wound vac was applied. Colchicine was started for the possibility of Behcet's syndrome but was stopped after one day when the patient's symptoms did not improve and pathology tests were negative. Dermatology recommended Valtrex for oral lesions. Patient's face and oral lesions began to resolve after the first dose. Vancomycin was discontinued due to high levels which started affecting the renal function. All cultures were negative and antibiotics were discontinued. Wound vacuum was removed from right ankle before discharge from the hospital. Patient followed up in the ambulatory clinic and wounds have healed very well.

## 10. Discussion

### 10.1. Herpes Ulcers

HSV 1 IgM blood titers were elevated in this patient. In addition to cellulitis at the site of the right ankle, the patient had history of small mouth ulcers but had never presented with skin lesions outside the mouth. Once patient was treated with acyclovir, mouth and facial lesions healed and ankle ulcer began to heal more quickly. Nongenital herpes simplex virus type 1 is a common infection usually transmitted during childhood via nonsexual contact or during adulthood through sexual contact. Oral mucosa or lips are usually involved in these infections. The primary lesion may range from being asymptomatic to being very painful, which can lead to poor nutritional intake. The recurrence of symptoms may be due to stress, fever, sun exposure, extreme temperatures, UV radiation, immunosuppression, or trauma. Other forms of herpes include herpetic keratitis infection of the eye, herpetic whitlow lesion found on hand or digits, and herpetic sycosis follicular infection. Treatment with oral acyclovir, valacyclovir, or famciclovir is effective for treatment and recurrence; see Table 1 (1).

### 10.2. Buruli Ulcer

 Buruli ulcer is a common ulceration found in tropical Africa, New Guinea, Malaysia, and Australia caused by* mycobacterium ulcerans*; see Table 1 (2). Mycobacterium ulcerans represents the third most common mycobacterial disease in intertropical areas. Clinical features consist of small mobile skin nodules that generally enlarge over days to weeks; lesions may resolve spontaneously but most will progress. Lesions generally are not painful and have no systemic inflammatory response. Eventually the papule breaks down at the center and forms an ulcer. Diagnosis consists of positive acid-fast bacilli culture from necrotic ulcer; PCR testing and punch biopsies can also be performed. Treatment of ulcer involves surgery to debride necrotic tissue.* Mycobacterium ulcerans* is generally sensitive to clarithromycin, rifampicin, and ethambutol.

### 10.3. Behcet's Syndrome

 Behcet's syndrome is a group of symptoms that occur together with unknown clear cause; see Table 1 (3). Clinical manifestations of Behcet's syndrome include oral ulceration, genital ulceration, uveitis, skin lesions including erythema nodosum, folliculitis, ulceration, hyperirritability or lethargy, arthritis, vasculitis, GI lesions, CV lesions, neurologic lesions, epididymitis, and family history. Diagnosis is based on clinical symptoms. Treatment includes use of colchicine and azathioprine and symptomatic treatment.

### 10.4. Erythema Multiforme

 Erythema multiforme is an acute and immune-mediated condition with the most common cause being drugs; see Table 1 (4). Drugs that cause reaction are NSAIDS, sulfonamides, antiepileptics, and antibiotics, but drug reaction is less than 10% of etiology. Clinical course is generally acute and self-limiting. Clinical symptoms include history of malaise, fever, myalgia, and cutaneous lesions involving round, erythematous, edematous papules surrounded by areas of blanching, which may appear as targetoid lesion with symmetrical distribution on extremities. There is not any available diagnostic test. Treatment in EM involves discontinuation of all inciting factors.

### 10.5. Syphilis

 Syphilis is a bacterial infection caused by the* Treponema pallidum*; see Table 1 (5). Syphilis is primarily transmitted through sexual contact and can also be transmitted through vertical transmission from mother to fetus. Primary syphilis is the first stage and typically presents clinically with a painless ulcer or chancre 14–28 days after initial exposure. Secondary syphilis presents with development of a flat maculopapular rash and can present with patches in mucosal membranes, headache, fatigue, lymphadenopathy, sore throat, and fever 6–8 weeks after development of chancre. Diagnosis is based on history and clinical symptoms, which can be diagnosed using VDRL and RPR. Treatment involves use of penicillin or doxycycline.

## 11. Conclusions 

This was an unusual presentation of herpes simplex virus infection. Patient was examined extensively but was only found to have HSV IgM and significant improvement of symptoms after starting antiviral therapy. After patient completed first course of antiviral therapy right ankle ulceration had delayed healing and once therapy was restarted the wound healing improved. Since she recently travelled to the United States, other diagnoses like Buruli ulcer and syphilitic ulcer took the center stage of diagnostic workup.

## Figures and Tables

**Table 1:** Multiple ulcer causing conditions with etiology and characteristics.

Condition	Etiology	Characteristics
(1) HSV ulcer	Herpes simplex virus type 1	Small, painful blister, vesicles, crusting, ulcer
(2) Buruli ulcer	*Mycobacterium ulcerans*	Painless nodule breaks down days to weeks forming ulcer
(3) Behcets ulcer	Autoimmune vasculitis	Oral apthous ulcers, cutaneous lesions with varying types
(4) Erythema multiforme (drug induced)	NSAIDS, sulfonamides, antiepileptics, antibiotics	Target lesion with central bullae, erythematous base
(5) Syphilitic ulcer	*Treponema pallidum*	Painless, circumferential ulcer
